# Supplementary material for: Acid cleavable biotin-alkyne improves sensitivity for direct detection of biotin labeled peptides in BONCAT analysis
Source: bioRxiv. 2024 Jul 17:2024.07.16.603801. Preprint. [Version 1] doi: 10.1101/2024.07.16.603801 (PMC11275937; doi:10.1101/2024.07.16.603801)
Supplement: Supplement 1 [file NIHPP2024.07.16.603801v1-supplement-1.pdf]

bioRxiv preprint doi: https://doi.org/10.1101/2024.07.16.603801; this version posted July 17, 2024. The copyright holder for this preprint (which was not certified by peer review) is the author/funder, who has granted bioRxiv a license to display the preprint in perpetuity. It is made available under aCC-BY 4.0 International license.

Supplementary Data:

All MS raw files are uploaded on ProteomExchange PXD053959.

bioRxiv preprint doi: https://doi.org/10.1101/2024.07.16.603801; this version posted July 17, 2024. The copyright holder for this preprint (which was not certified by peer review) is the author/funder, who has granted bioRxiv a license to display the preprint in perpetuity. It is made available under aCC-BY 4.0 International license.

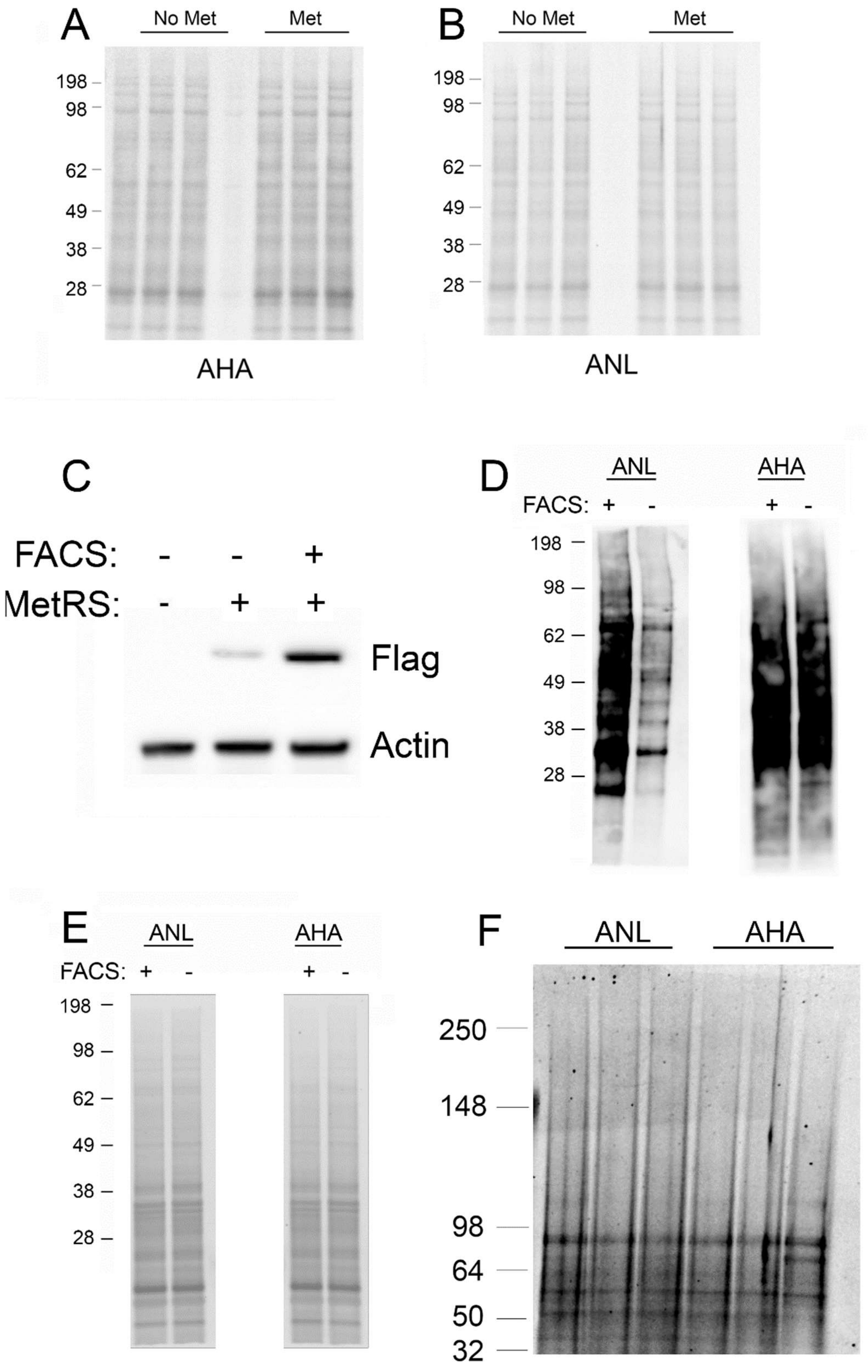

Supplementary Figure 1

bioRxiv preprint doi: https://doi.org/10.1101/2024.07.16.603801; this version posted July 17, 2024. The copyright holder for this preprint (which was not certified by peer review) is the author/funder, who has granted bioRxiv a license to display the preprint in perpetuity. It is made available under aCC-BY 4.0 International license.

Figure S1. **A**, Total protein content of AHA samples in Fig1A using a protein stain. **B**, Total protein content of ANL samples in Fig1B using a protein stain. **C,** Immunoblot analysis of mMetRS transfected cells with and without FACS using a Flag tag antibody. **D**, Immunoblot analysis of ANL and AHA labeled cells with and without FACS probed with streptavidin-HRP. **E**, Total protein content of samples in D using a protein stain. **F**, Total protein content of samples in Fig. 1D using a protein stain.

bioRxiv preprint doi: https://doi.org/10.1101/2024.07.16.603801; this version posted July 17, 2024. The copyright holder for this preprint (which was not certified by peer review) is the author/funder, who has granted bioRxiv a license to display the preprint in perpetuity. It is made available under aCC-BY 4.0 International license.

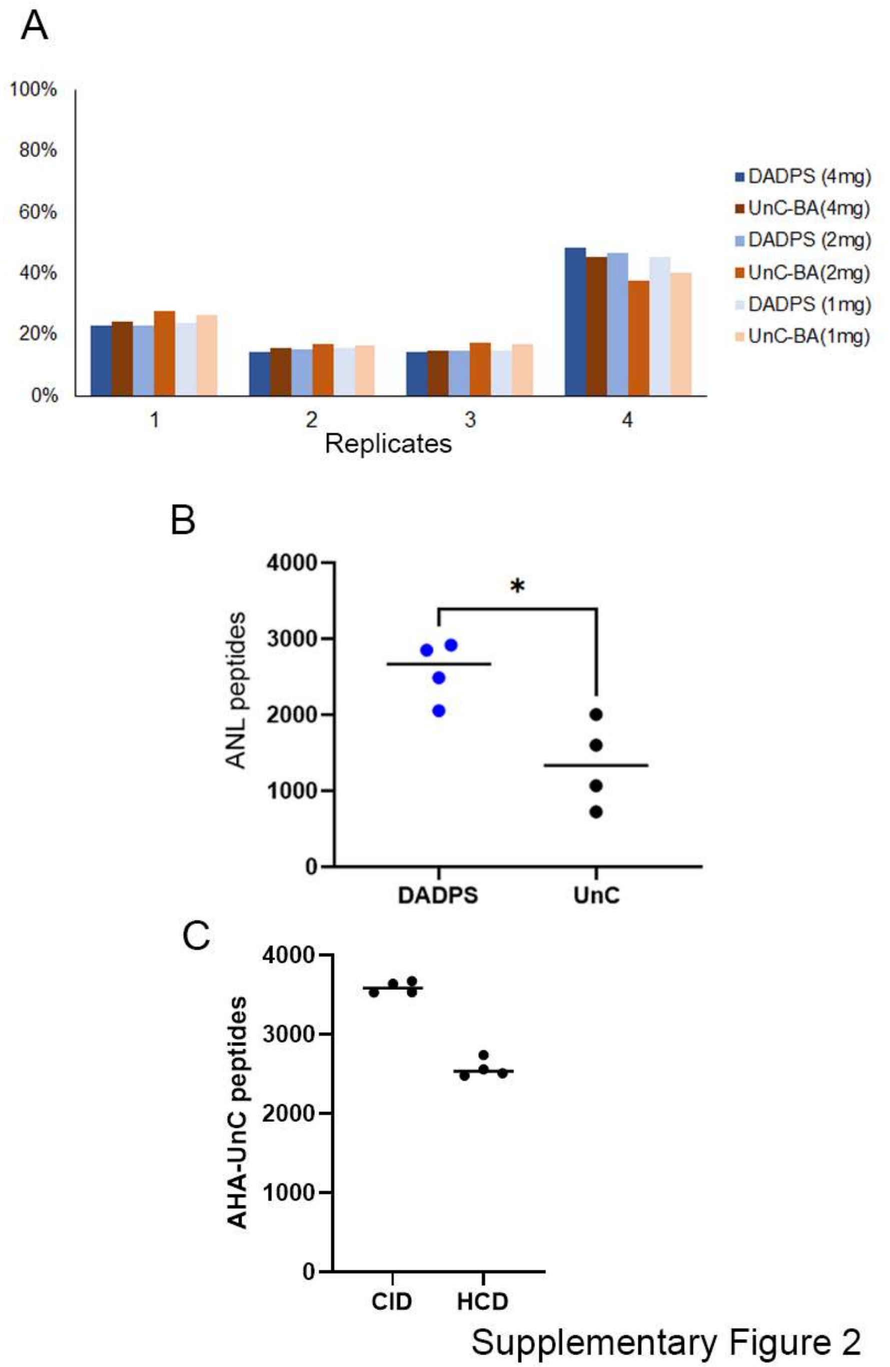

Figure S2. **A,** The number of replicates an AHA-BA peptide was identified by in Figure 2A. **B,** The number of ANL-BA peptides identification using either DADPS or UnC. **C,** One UnC-AHA sample was injected on an Orbitrap Fusion Lumos mass spectrometer (Thermo Fisher Scientific) using either an HCD or CID fragmentation in the ion trap.
